# Home-based lifestyle intervention for breast cancer survivors: A surprising improvement in the quality of life during the first year of COVID-19 pandemic

**DOI:** 10.1371/journal.pone.0296163

**Published:** 2024-01-02

**Authors:** Denise Vagnini, Valentina Natalucci, Sara Moi, Luciana Vallorani, Alice Pietrelli, Andrea Rocco Panico, Carlo Ferri Marini, Francesco Lucertini, Giosuè Annibalini, Davide Sisti, Marco Bruno Luigi Rocchi, Vincenzo Catalano, Emanuela Saita, Rita Emili, Elena Barbieri

**Affiliations:** 1 Department of Psychology, Università Cattolica del Sacro Cuore, Milan, Italy; 2 Department of Biomolecular Sciences, Division of Exercise and Health Sciences, University of Urbino Carlo Bo, Urbino, Italy; 3 Medical Oncology Unit, AST Pesaro-Urbino, Santa Maria della Misericordia Hospital Urbino, Urbino, Italy; PLOS ONE, UNITED KINGDOM

## Abstract

**Introduction:**

The COVID-19 pandemic induced an extraordinary impact on public mental health to a degree not completely understood, especially in vulnerable populations such as breast cancer (BC) survivors. In this study, we described the short- (after 3-month) and long- (after 12-month) term effects of a multidisciplinary home-based lifestyle intervention in Italian women BC survivors during the first year of COVID-19 pandemic.

**Materials and methods:**

In total, 30 Italian BC survivors with risk factors for recurrence took part in the ongoing MoviS trial (protocol: NCT 04818359). Between January 2020 and January 2021, a 3-month lifestyle intervention based on psychological counseling, nutrition, and exercise was carried out. Participants were asked to fill out psychological questionnaires for the assessment of quality of life (QoL) indicators (European Organization for Research and Treatment of Cancer QoL, EORTC-QLQ-C30) and psychological health measures such as fatigue (Brief Fatigue Inventory, BFI), distress (Distress Thermometer, DT and Psychological Distress Inventory, PDI), cancer-related fatigue (Verbal Rating Scale, VRS), and mood states (Profile of Mood States Questionnaire, POMS). IBM SPSS Statistical Software version 27.0 and R Project for Statistical Computing version 4.2.1 were used to process data. All participants were assessed at four time points: T0 (baseline), T1 (3-month), and follow-up at T2 and T3 (6- and 12-month, respectively) to measure primary (quality of life indicators) and secondary (psychological health) outcomes. Friedman non parametric test and Wilcoxon signed rank test (with Bonferroni correction) were conducted to investigate the statistically significant differences in psychometric scores and between assessment times.

**Results:**

Compared to baseline (T0), at T1 most of the QoL indicators (i.e., symptoms of fatigue and general health) were improved (*p* < 0.017) with the exception of a worsening in participants’ social functioning ability. Also, perception of severity of fatigue, distress, cancer-related fatigue, depression, and anger enhanced. Compared to baseline (T0), at T3 we mainly observed a stable condition with T0-T1 pairwise comparison, however other secondary outcomes (i.e., fatigue mood state, confusion, and anxiety) significantly improved.

**Discussion:**

Our preliminary findings support the proposal of this lifestyle intervention for BC survivors. Despite the home-confinement due to the COVID-19 pandemic, the intervention surprisingly improved QoL indicators and psychological health of the participants.

## Introduction

After nearly three years since the lockdown due to the “Coronavirus Disease 2019” pandemic (COVID-19), the empirical findings suggest a global post COVID-19 condition prevalence of approximately 43% and the regional prevalence estimate in Europe is approximately 44%. Moreover, data suggests that female adults had both higher prevalence and risk of having post COVID-19 conditions than male adults (49% vs 37%) [1]. In these years, while evidences on the viral transmission and pathogenesis have been well documented in the scientific literature, little is known about the long-term physical and psychological consequences (e.g., experiences of distress, psychiatric disorders, and engagement in pandemic-related health behaviors), especially in vulnerable populations such as patients with breast cancer (BC). Despite the pandemic, while the cancer death rate continued to decline from 2019 to 2020 (by 1.5%) contributing to a 43% overall reduction from 1989 through 2020, cancer incidence increased for BC [2]. This progress in the incidence of BC has led to increasing attention to the management of certain aspects related to diagnosis and treatment. In fact, the BC diagnosis and treatment have a strong impact on women’s emotional health and quality of life (QoL) [3]. It has been demonstrated that cancer treatments (i.e., surgery and neo/adjuvant therapies) could lead to short- and long-term problems that can involve not only the physical but also the psychological sphere (e.g., cancer-related-fatigue, perception of a lack of psycho-physical self-integrity, or psycho-physical impairment such as anxiety and depressive symptoms, distress, and social/relational isolation) with a negative impact on cancer survivors’ QoL [4, 5]. Patients with BC are not only characterized by increased vulnerability because of modified living, but they also had to adapt to delays and changes in clinical procedures due to the COVID-19 emergency [6]. Indeed, patients with BC appeared to be more vulnerable to worse outcomes of infection not only in terms of clinical care [7], but also on the physiological and psychological responses, which have been negatively modulated with escalating symptoms and diagnoses of depression and anxiety [8]. In general, during the first year of the COVID-19 pandemic, patients with cancer have experienced two barriers: on the one hand, clinical barriers with the deprivation of diagnostic procedures and screening programs; on the other, barriers to supportive interventions (e.g., exercise delivery programs, psychological and nutritional counseling) [9–13].

In a holistic view of care, the outcomes of BC medical treatments could impact different areas of life. BC patients’ health should be treated as a continuum of bio-psycho-social interconnections [14], within the unique concept of global QoL.

The World Health Organization defines QoL as *“The condition of life resulting from the combination of the effects of a complete range of factors such as those determining health*, *happiness including comfort in the physical environment and a satisfying occupation*, *education*, *social and intellectual attainments*, *freedom of action*, *justice*, *and expression”* [15]. Although there are different meanings of QoL, it represents an important outcome measure in BC clinical investigations and survivorship studies [16].

As shown in the literature, this condition can be profoundly and negatively affected by extreme events such as pandemics. General research on the impact of previous epidemics and pandemics (e.g., SARS, H1N1, and Ebola) on mental health suggests elevated and long-lasting symptoms [17]. Considering that similar symptoms have also been found for the latest COVID-19 pandemic both in the general population and in the fragile one such as patients with BC, it is necessary to implement strategies that can mitigate these effects. About that, an intervention program for a healthy and active lifestyle with psychological support can profoundly affect both short- and long-term health and QoL and could represent an important non-pharmacological intervention that might positively influence cancer survival [18–20]. Emerging evidence confirms the safety, feasibility, and effectiveness of specific exercise training in mitigating cancer treatments’ side-effects, such as musculoskeletal pain and fatigue, and improving physical, cardiometabolic, emotional wellbeing, and global health-related QoL (HRQoL) [21]. In this regard, attention to modifiable behaviors—such as exercise and nutrition—has grown in recent years, with particular consideration to the strategies to be adopted in the clinical outcomes of BC survivors [22–27] even more during the COVID-19 era. In the Italian context, the growing literature on the population with BC suggests the effectiveness of a multidisciplinary approach to achieve and maintain HRQoL in the long term. This represents, today, a real challenge in the daily clinical practice because dealing with oncological disease requires an integration between scientific and clinical fields to decline the work in an “active” way by intervening on reality in a concrete and translational manner [24, 28–30].

In this study, we described the short- (after 3-month) and long- (after 12-month) term effects of a multidisciplinary home-based lifestyle intervention from women with BC of the ongoing MoviS (Movement and health beyond care) trial [31] during the first year of COVID-19 pandemic.

Our study has two main aims on the effects of the intervention: the first is the analysis of QoL indicators (primary outcomes), and the second is the evaluation of the psychological health (secondary outcomes) of BC survivors.

Specifically, we hypothesized that, despite the dramatic pandemic context in evolution, the proposed home-based lifestyle intervention could improve primary (indicators of QoL) and secondary (psychological health) outcomes in BC survivors’ women with a high risk of recurrence in the short-term (3-month post lifestyle intervention) (Hypothesis 1) and in the long-term (12-month follow-up assessment) (Hypothesis 2) with stable outcomes over time.

## Materials and methods

### Study design and amended

Initially, study design (Clinical trial Identifier NCT 04818359) [31] was projected as an open randomized controlled trial with two parallel arms (with a 1:1 ratio). Patients taken care of the oncology clinic of the Department of Medical Oncology of the Urbino Hospital (PU, Italy) were pre-recruited and carefully informed about the project. Written informed consent was obtained from all individual participants included in the study. The Human Research Ethics committee of the University of Urbino Carlo Bo approved the study (Protocol N. 21 of 10 July 2019). Due to the imposed COVID-19 pandemic restrictions, the study protocol was amended and approved by the Human Research Ethics committee of the University of Urbino Carlo Bo (amended Protocol N. 29 of 22 April 2020). As reported in Natalucci et al., 2021 [19], the forced changes in the study protocol made the difference on cardiometabolic parameters between intervention arm and control arm interventions negligible, providing similar adaptations between groups. As described in the statistical paragraph, using the Mann-Whitney U test, psychological differences between the two arms were also studied, and results showed the same trend highlighting no significant differences in the short-term period (i.e., after 3-month of lifestyle intervention). Therefore, due to the lack of meaningful differences the two arms were combined.

### Population

Participants have been identified by the oncology clinic of the Medical Oncology Department of the Urbino Hospital (PU) in the Marche region (central Italy), and they were recruited for the study if they met the following inclusion criteria: after surgery (maximum 12-month) and chemotherapy and/or radiotherapy adjuvant treatments (minimum 6 months); stage 0 to III BC without evidence of metastases or diagnosed recurrences at recruitment in follow-up assessed by medical records; aged 30–70 years; non-physically active, namely participants must be not regularly active (assessed by the International Physical Activity Questionnaire Short Form, IPAQ-SF) [32, 33] for at least 6 months (i.e., not engaged in at least 60 min/week of structured exercise during the previous 6 months); with a risk factor for recurrence. As reported in previous studies [34], the risk factors for recurrence were the presence of at least 1 of the following conditions: body mass index (BMI) at diagnosis ≥ 25 kg/m2; testosterone ≥ 0.4 ng/mL; serum insulin ≥ 25 μU/mL (170 pmol/L); metabolic syndrome (at least 3 of the following 5 factors): (a) glycemia ≥ 100 mg/dL (6.05 mmol/L); (b) triglycerides ≥ 150 mg/dL (1.69 mmol/L); (c) HDL-C < 50 mg/dL (1.29 mmol/L); (d) waist circumference ≥ 80; (e) blood pressure ≥ 130/85 mmHg.

Exclusion criteria were: disabling pneumological, cardiological, neurological, orthopedic comorbidities, or mental illnesses that prevent the exercise performance; treatment with beta blockers, non-dihydropyridine calcium channel blockers or amiodarone, due to their potential effect on heart rate response to exercise; treatment with antidepressant drugs.

### Sample collection

Recruitment occurred in January 2020 from the Santa Maria della Misericordia Hospital of Urbino (PU) in the Marche region (central Italy). Between January 2020 and January 2021, a trained psycho-oncologist collected data through face-to-face questionnaires at the following four time points: T0 (baseline), T1, T2 and T3 (after 3-, 6- and 12- month, respectively). Participants’ general and medical characteristics were collected at T0 by an oncologist.

### Intervention

The lifestyle intervention took place after surgery and upon completion the primary treatments (post chemo- or radio-therapy adjuvant). As previously described [19], the original lifestyle intervention protocol had participants randomized into intervention and control arm. Briefly, the lifestyle intervention comprised two phases. The first (study enrollment) in which participants of both arms received structured meetings lasting about one hour (45 min in group setting and 15 personalized minutes) with focus on current exercise guidelines and on Mediterranean diet, respectively. In addition, both arms received psychological support by the psycho-oncologist and registration on the DianaWeb platform. The DianaWeb is a community-based participatory research that offers to Italian women, with a diagnosis of BC, an interactive website (www.dianaweb.org). This platform was used to provide all participants evidence-based diet/lifestyle recipes, exercises, and recommendations to manage improvement in daily routine [35, 36]. The second (intervention phase), in which participants of the intervention arm received a 3-month of MoviS training that consisted of: on-site (2 sessions per week) and remotely (1 session per week) supervised aerobic exercise training program having progressive increases in exercise intensity (from 40% to 70% of heart rate reserve [HRR]) and duration (from 20 to 60 min). In the MoviS training, exercise intensity and duration were gradually increased to reach and exceed the recommended quality (exercise intensity) and quantity (volume) of aerobic exercise [37, 38]. Both remotely and on-site supervised training sessions consisted of aerobic exercise (i.e., walking, running, or cycling). On-site supervised sessions were performed in a gym using a treadmill or stationary bikes, whereas the remotely supervised sessions were performed both indoors and/or outdoors according to participants’ possibilities and preferences. Regardless of the exercise modality and supervision, the heart rate responses were monitored using heart rate monitors (ONRHYTHM 110, Kalenji) which were given to the participants at the training sessions. The external exercise intensities (e.g., bike wattage or treadmill speed and grade) were adjusted to maintain the target heart rate (HR) throughout each training session to account for several physiological adjustments to prolonged aerobic exercise.

The HR values corresponding to the desired %HRR used to prescribe aerobic exercise intensity were calculated as follows: (maximal HR—pre-exercise HR) x desired percentage + pre-exercise HR. Pre-exercise HR was measured after 5-min standing, while maximal HR was predicted as proposed by Gellish et al. [39].

Due to the imposed COVID-19 pandemic restrictions, the first phase has not undergone changes while the second phase has undergone the following changes. Participants in the intervention arm started the MoviS training following the original protocol but, from the 4^th^ week of MoviS training supervision was adapted to solely remotely supervised exercise (3 sessions per week). Remote supervision was performed weekly, using phone calls from the exercise specialist, who provided the weekly exercise prescription and personalized feedback according to the training logs. The participants could choose the exercise mode in relation to the restrictions and the availability of tools such as treadmills or bikes at home, while monitoring and prescription by the trainer were always based on the prescribed target HR.

During the intervention, participants in the control group received usual care, as described above by structured meetings they received lifestyle recommendations (diet recipes, exercises, and suggestions) to manage improvement in daily routine based on nutrition and physical activity guidelines [35–37]) and were remotely supported, individually instructed and monitored using group chat messages in a random weekday and time. Briefly, message content included encouragement, support, and practical tips regarding healthy lifestyle.

### Assessments

Psychometric measures were collected (at T0, T1, T2, and T3) by a psycho-oncologist through validated questionnaires that were filled out by the participants in 15–20 minutes.

### Primary outcomes: Quality of life indicators

European Organization for Research and Treatment of Cancer Quality of Life (EORTC-QLQ-C30) consists of a global health and quality of life scale (GHS) rated on a 7-point Likert (from 1 = very poor, to 7 = excellent) that is based on two questions about physical condition and overall QoL, and nine multi-item scales on a 4-point Likert (from 1 = not at all, to 4 = very much) that reflect the multidimensionality of the QoL construct and incorporate five Functional Scales (FS: physical, role, cognitive, emotional, and social), and three Symptom Scales (SS: fatigue, pain, and nausea and vomiting) [40]. The remaining single items assess additional symptoms commonly reported by cancer patients (e.g., dyspnea, appetite loss, sleep disturbance, constipation, and diarrhea), as well as the perceived financial impact of the disease and treatment. In this specific study we considered only the five subscales indicative of psychological symptoms (i.e., FS: role, cognitive, emotional, and social; SS: fatigue), and the GHS. Examples of items are: “Have you had difficulty concentrating on things, like reading a newspaper or watching television?”; “Has your physical condition or medical treatment interfered with your social activities?”. The scale and subscale structures meet the minimal standard for reliability (Cronbach’s alpha coefficient ≥ 0.70). In this study the Italian version [41] was used and we observed Cronbach’s alpha ratings between the minimum threshold and 0.876.

### Secondary outcomes: Psychological health

#### Brief Fatigue Inventory (BFI)

BFI consists of 9 items rated on 11-point Likert scale. The first three items indicate the severity of general fatigue experienced by participants (from 0 = no fatigue, to 10 = the worst level of fatigue that can be experienced); while items 4 to 9 indicate how much the perceived fatigue has interfered with the element or activity in question (i.e., general daily activity, the mood, the ability to walk, the ability to work at home and outside, the interpersonal relationships, and the ability to have fun), from 0 = it did not interfere with this, to 10 = it interfered completely with this activity. Examples of items are: “Please rate your general activity”; “Please rate your relations with other people”. The validity and reliability of the original BFI have been established with a strong internal consistency coefficient of 0.96 [42]; in the present study it was 0.906.

#### Distress Thermometer (DT)

DT consists of a single-item rated on a 11-point Likert scale (from 0 = no distress, to 10 = extreme distress; with a midpoint of 5 = moderate distress) which measures the subjective emotional distress experienced during the last week and evaluates it through a visual analog scale with the shape of a thermometer that is extremely user-friendly. The item is: “Please circle the number 0 to 10 that best describes how much distress you have been experiencing in the past week including today” [43].

#### Psychological Distress Inventory (PDI)

PDI consists of a self-administered 13-item questionnaire rated on a 5-point Likert scale (from 1 = not at all, to 5 = extremely), ranging from 13 to 65 to indicate the present mood of the patient, with 1 week reference period. It measures the general emotional lability of cancer patients and more specifically disorders tied to adjustment: (a) reactive anxiety to cancer, such as inner tension and worry; (b) reactive depression; and (c) emotional reactions to changes in the body image, and disturbances in the interpersonal context. Examples of items are: “In the last week have you felt worthless?”; “In the last week has your interest in the world that surrounds you diminished?”. Internal consistency was reported at 0.84 to 0.88 Cronbach’s alpha ratings [44, 45], and in the current study was 0.874.

#### Verbal Rating Scale (VRS)

It is composed of a single-item rated on a 6-point Likert Scale (from 1 = none, to 6 = very severe) to specifically evaluate the cancer-related fatigue (CRF). The item is: “Choose below the level of fatigue you are experiencing” [46].

#### Profile of Mood States Questionnaire (POMS)

The Italian version consists of 65 items rated on a 5-point Likert scale (from 0 = not at all, to 4 = extremely) for the assessment of mood states. It is divided into six subscales: depression, anger, fatigue, confusion, strength/vigor, and anxiety. Examples of items are: “Describe how you feel right now: confused”; “Describe how you feel right now: nervous”. Internal consistency was reported at 0.63 to 0.96 Cronbach’s alpha ratings [47, 48], in the present study subscales’ coefficients were between 0.786 and 0.959.

### Statistical analysis

Data were analyzed using IBM SPSS Statistical Software version 27.0 and R Project for Statistical Computing version 4.2.1. The participants who completed the assessments at every time point (T0, T1, T2, and T3) were included in the analysis. In this way, we were able to answer hypothesis 1 (i.e., the lifestyle intervention was effective in the immediate post-intervention: T0-T1 comparison) and hypothesis 2 (i.e., the intervention’s efficacy on primary and secondary outcomes was highlighted over time: comparison between baseline and T3).

Missing value analysis was conducted to study missing data in terms of frequencies and percentages. Incomplete partial responses were retained, and no attempt was made to replace missing data.

Cronbach’s alpha coefficient was used to measure internal consistency of each psychometric measure with acceptable values ≥ 0.65 for self-report questionnaires [49].

Normality of distributions and extreme values were studied using visual analysis on Q-Q plots and the Shapiro-Wilk test, considering a normal distribution for *p* > 0.05.

Descriptive statistics (i.e., frequency, mean, and standard deviation) were performed to describe participants’ demographics, clinical, and psychological characteristics.

A graphical display of a non-parametric correlation matrix, based on Spearman’s R, ordered according to hierarchical clustering, was obtained using the corrplot R package. Considering the small sample size and the non-normal distribution of the data, non-parametric tests have been used. We primarily assessed the absence of psychological differences at T0 between the intervention arm and the control arm using the non-parametric Mann-Whitney U test, fixing the significant threshold at the standard level of *p* < 0.05. Then, as previously mentioned in the study design paragraph, we compared the two arms longitudinally without finding significant differences on psychological variables, as well as anthropometric and cardiometabolic data reported in Natalucci et al., 2021 [19]. The analysis of these data using a multivariate general linear model showed a very small effect size (F = 0.664, *p* = 0.665, Cohen’s f = 0.09) of the interaction between time (pre- and post-intervention) and study arm (intervention and control) on several representative parameters (BMI, glycemia, triglycerides, LDL, and maximal oxygen uptake).

Therefore, the analyzes were conducted considering all the enrolled participants as a single group. The total sample was composed of N = 30 women with a previous diagnosis of BC. For the primary outcomes (QoL indicators), we calculated the effect size using t-tests for means; for the difference between two dependent means (matched pairs at T0 and T1), with the sample size reported in the paper (i.e., N = 30), we observed a Cohen’s [50] effect size equal to 0.464 (value close to medium effect). Efficacy of the intervention on primary (QoL indicators) and secondary (psychological health) outcomes was assessed with the non-parametric Friedman’s test (for k > 2 related-measures).

Mean, standard deviation, significance, and Kendall’s W effect size (ranging from 0 = no relationship, to 1 = perfect relationship) were provided [50, 51].

To verify our hypothesis 1 (i.e., efficacy of the lifestyle intervention at T1, on primary and secondary outcomes) and hypothesis 2 (i.e., efficacy over time, at T3), multiple post hoc comparisons with non-parametric Wilcoxon signed rank test (for two related-measures: T0-T1; T0-T2; T0-T3) were then performed using Bonferroni correction (critical level *p* < 0.017) to determine where there was significant change. Effect sizes for r = Z/√N, in which N represents the number of observations [52], were provided using Cohen’s criteria for interpretation [50].

## Results

**Fig 1:**
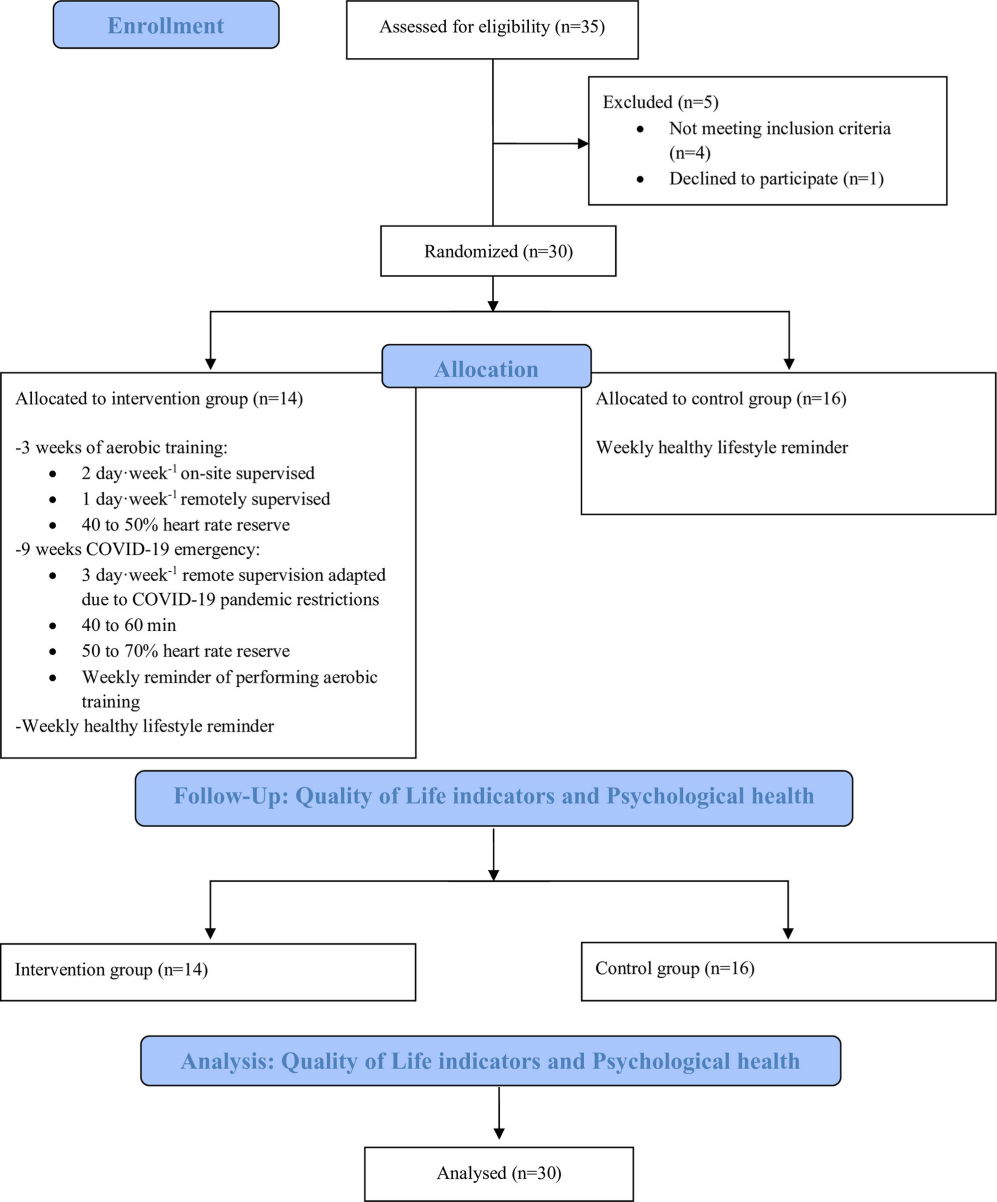
Study flowchart.

In total, data from 30 study participants were analyzed. The study flowchart (Fig 1) provides further details. Missing value analysis showed that missing data ranged from 3.3% (n = 1 item), to 23.3% (n = 7 items).

### Sample characteristics

At T0, participants (N = 30 BC survivors) age and time since diagnosis were 53.6 ± 7.6 years (range 39–69) and 10.37 ± 2.87 months (range 4–12), respectively. Baseline participants characteristics are described in Table 1. At 12-month post-intervention, no cardiovascular incidents and no relapses were observed.

**Table 1:** Participants baseline characteristics (N = 30).

Variables	*n* (%)
Marital status	
Single	6 (20)
Married	21 (70)
Divorced	3 (10)
Educational level	
Elementary school	4 (13.33)
Secondary school	6 (20)
College degree	0 (0)
University degree	20 (66.67)
Working status	
Employee	27 (90)
Sick leave	1 (3.33)
Retired	2 (6.67)
Side affected by carcinoma	
Right	15 (50)
Left	15 (50)
Surgery	
Quadrantectomy	26 (86.67)
Mastectomy	3 (10)
Lumpectomy	1 (3.33)
Radiotherapy	
Yes	24 (80)
No	6 (20)
Chemotherapy	
Yes	13 (43.33)
No	17 (56.67)
Hormonal therapy	
Tamoxifen	8 (26.67)
Aromatase inhibitors	16 (53.33)
No	6 (20)

**Fig 2:**
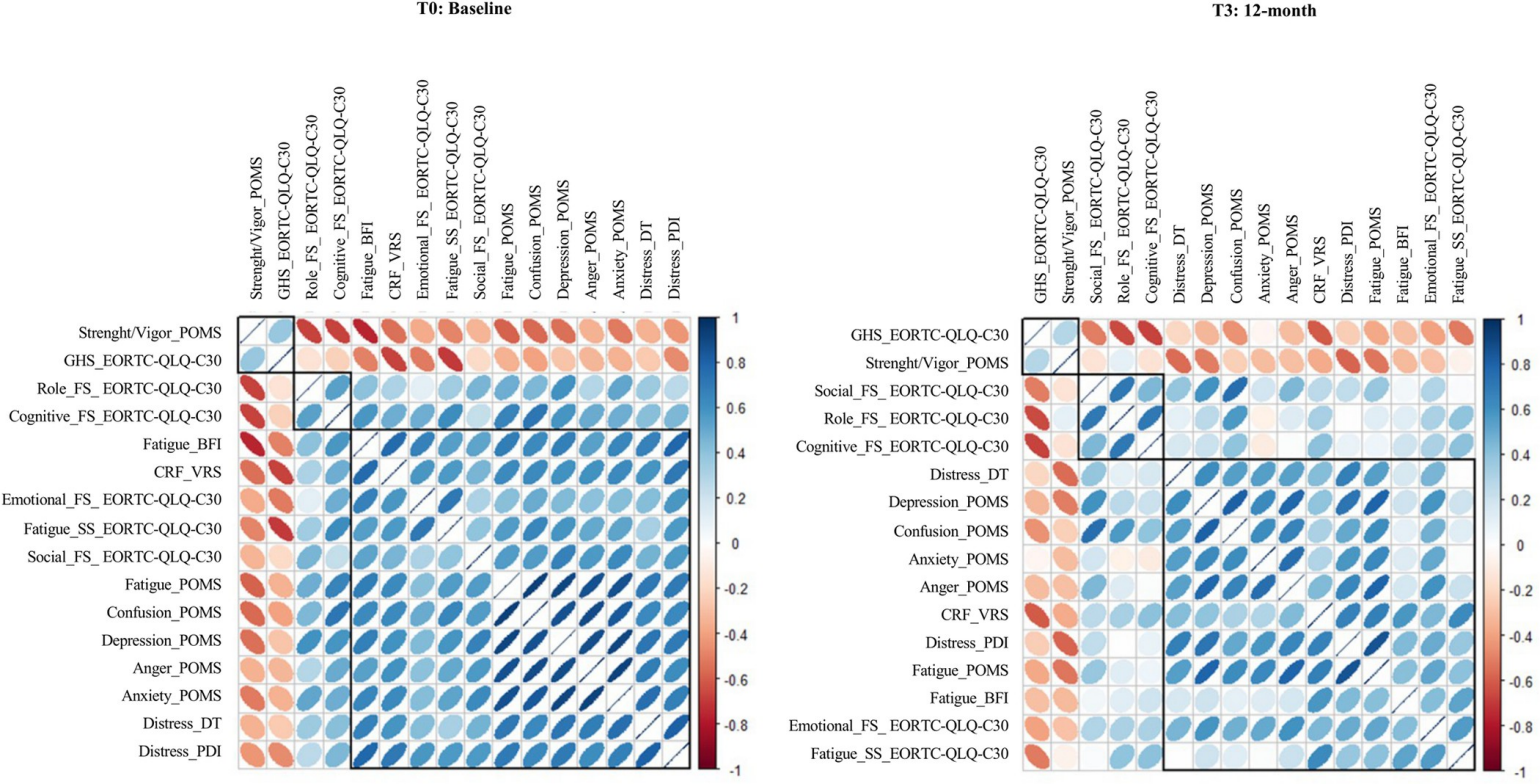
Corrplot at T0 (baseline) and T3 (12-month) of psychometric variables. Abbreviations: EORTC-QLQ-C30, European Organization for Research and Treatment of Cancer Quality of life questionnaire; FS, Functional scales; SS, Symptom scales; GHS, Global Health Status; BFI, Brief Fatigue Inventory; DT, Distress Thermometer; PDI, Psychological Distress Inventory; VRS, Verbal Rating Scale; CRF, Cancer-related fatigue; POMS, Profile of Mood States Questionnaire.

Considering both primary and secondary outcomes at T0 and T3, Fig 2 displays the graphical non-parametric correlation matrix (Spearman’s R) ordered according to hierarchical clustering.

### Longitudinal intra-group (N = 30) comparisons over a year

Table 2 summarizes Friedman’s test results and provides descriptive statistics for all psychometric measures (primary and secondary outcomes). Pairwise comparisons (T0-T1; T0-T2; and T0-T3) with Bonferroni correction (*p* < 0.017) for primary and secondary outcomes are presented in Table 3.

**Table 2:** Friedman’s test for all psychometric measures.

Measures (range)	T0 $\bar{x}$ ± SD	T1 $\bar{x}$ ± SD	T2 $\bar{x}$ ± SD	T3 $\bar{x}$ ± SD	χ^2^ (*p*)^a^	Effect Size (Kendall’s W)^b^
**Primary outcomes**						
**EORTC-QLQ-C30**						
FS-Role (1–4)	1.23 ± 0.46	1.07 ± 0.22	1.13 ± 0.23	1.25 ± 0.52	6.73 (*p* = 0.081)	0.08
FS-Cognitive (1–4)	1.55 ± 0.83	1.20 ± 0.37	1.36 ± 0.41	1.34 ± 0.51	8.63 (*p* = 0.035)*	0.10
FS-Emotional (1–4)	1.73 ± 0.56	1.54 ± 0.55	1.54 ± 0.47	1.54 ± 0.48	2.38 (*p* = 0.498)	0.03
FS-Social (1–4)	1.57 ± 0.70	1.00 ± 0.01	1.02 ± 0.10	1.05 ± 0.28	37.24 (*p* < 0.001)**	0.45^x^
SS-Fatigue (1–4)	1.75 ± 0.69	1.33 ± 0.42	1.45 ± 0.40	1.29 ± 0.35	15.38 (*p* = 0.002)*	0.18
GHS (1–7)	4.91 ± 1.03	5.52 ± 0.86	5.76 ± 0.85	5.56 ± 1.03	18.00 (*p* < 0.001)**	0.22
**Secondary outcomes**						
**BFI (0–10)**						
Fatigue	3.14 ± 1.92	1.92 ± 1.06	1.97 ± 1.53	2.03 ± 1.24	11.23 (*p =* 0.011)*	0.13
**DT (0–10)**						
Distress	5.08 ± 3.05	3.08 ± 2.33	3.96 ± 2.51	3.32 ± 2.58	16.65 (*p* < 0.001)**	0.21
**PDI (1–5)**						
Distress	2.21 ± 0.62	2.04 ± 0.52	1.89 ± 0.62	1.87 ± 0.70	10.43 (*p =* 0.015)*	0.14
**VRS (1–6)**						
CRF	3.38 ± 0.97	2.38 ± 1.32	2.62 ± 1.28	2.81 ± 1.03	9.90 (*p* = 0.019)*	0.12
**POMS (0–4)**						
Depression	0.99 ± 0.98	0.42 ± 0.55	0.31 ± 0.52	0.33 ± 0.48	22.74 (*p* < 0.001)**	0.32^x^
Anger	1.41 ± 1.12	0.80 ± 0.74	0.65 ± 0.63	0.53 ± 0.57	18.59 (*p* < 0.001)**	0.26
Fatigue	1.47 ± 1.06	0.90 ± 0.80	0.76 ± 0.67	0.62 ± 0.54	12.82 (*p =* 0.005)*	0.18
Confusion	1.39 ± 0.79	1.05 ± 0.54	0.88 ± 0.61	0.76 ± 0.61	19.08 (*p* < 0.001)**	0.27
Strength/Vigor	2.02 ± 0.65	2.49 ± 0.71	2.77 ± 0.79	2.23 ± 0.67	18.34 (*p* < 0.001)**	0.26
Anxiety	1.41 ± 0.81	0.92 ± 0.67	0.89 ± 0.66	0.80 ± 0.51	12.49 (*p =* 0.006)*	0.17

Abbreviations: EORTC-QLQ-C30, European Organization for Research and Treatment of Cancer Quality of life questionnaire; FS, Functional Scales; SS, Symptom scales; GHS, Global Health Status; BFI, Brief Fatigue Inventory; DT, Distress Thermometer; PDI, Psychological Distress Inventory; VRS, Verbal Rating Scale; CRF, Cancer-related fatigue; POMS, Profile of Mood States Questionnaire.

^a^ χ^2^ test: the degrees of freedom are 3 for all the values.

^b^ Effect Size (Kendall’s W): |0.1| ≤ W < |0.3| small effect; |0.3| ≤ W < |0.5| medium effect^x^; W ≥ |0.5| = large effect

*p < 0.05

**p < 0.001

**Table 3:** Post hoc Wilcoxon signed rank test: Pairwise comparisons.

Measures (range)	T0-T1 (baseline– 3-month)		T0-T2 (baseline– 6-month)		T0-T3 (baseline– 12-month)	
	Z (*p*)	Effect Size (r)^a^	Z (*p*)	Effect Size (r)^a^	Z (*p*)	Effect Size (r)^a^
**Primary outcomes**						
**EORTC-QLQ-C30**						
FS-Role (1–4)	NA	NA	NA	NA	NA	NA
FS-Cognitive (1–4)	–2.42 (*p* = 0.048)	–0.31^x^	–1.34 (*p* = 0.537)	–0.18	–1.79 (*p* = 0.222)	–0.23
FS-Emotional (1–4)	NA	NA	NA	NA	NA	NA
FS-Social (1–4)	–3.50 (*p* = 0.003)*	–0.45^x^	–3.27 (*p* = 0.003)*	–0.43^x^	–3.37 (*p* = 0.003)*	–0.44^x^
SS-Fatigue (1–4)	–3.14 (*p* = 0.006)*	–0.41^x^	–1.94 (*p* = 0.159)	–0.25	–2.89 (*p* = 0.012)*	–0.38^x^
GHS (1–7)	–3.14 (*p* = 0.006)*	–0.41^x^	–3.54 (*p* = 0.003)*	–0.46^x^	–3.00 (*p* = 0.009)*	–0.39^x^
**Secondary outcomes**						
**BFI (0–10)**						
Fatigue	–2.99 (*p* = 0.009)*	–0.39^x^	–2.87 (*p* = 0.012)*	–0.38^x^	–3.04 (*p* = 0.006)*	–0.40^x^
**DT (0–10)**						
Distress	–4.23 (*p* = 0.003)*	–0.55^xx^	–1.88 (*p* = 0.180)	–0.25	–3.02 (*p* = 0.006)*	–0.40^x^
**PDI (1–5)**						
Distress	–1.29 (*p* = 0.594)	–0.17	–2.57 (*p* = 0.030)	–0.34^x^	–2.95 (*p* = 0.009)*	–0.39^x^
**VRS (1–6)**						
CRF	–2.78 (*p* = 0.015)*	–0.39^x^	–2.39 (*p* = 0.051)	–0.33^x^	–2.80 (*p* = 0.015)*	–0.39^x^
**POMS (0–4)**						
Depression	–3.24 (*p* = 0.003)*	–0.42^x^	–2.89 (*p* = 0.012)*	–0.38^x^	–3.43 (*p* = 0.001)*	–0.46^x^
Anger	–2.90 (*p* = 0.012)*	–0.38^x^	–2.83 (*p* = 0.015)*	–0.38^x^	–2.96 (*p* = 0.009)*	–0.40^x^
Fatigue	–2.42 (*p* = 0.024)	–0.32^x^	–3.17 (*p* = 0.006)*	–0.42^x^	–3.64 (*p* = 0.003)*	–0.49^x^
Confusion	–1.50 (*p* = 0.399)	–0.20	–3.06 (*p* = 0.006)*	–0.41^x^	–3.45 (*p* = 0.003)*	–0.46^x^
Strength/Vigor	–2.01 (*p* = 0.132)	–0.26	–3.29 (*p* = 0.003)*	–0.44^x^	–1.58 (*p* = 0.345)	–0.21
Anxiety	–2.72 (*p* = 0.021)	–0.35^x^	–2.71 (*p* = 0.021)	–0.36^x^	–3.05 (*p* = 0.006)*	–0.41^x^

Abbreviations: EORTC-QLQ-C30, European Organization for Research and Treatment of Cancer Quality of life questionnaire; FS, Functional Scales; SS, Symptom scales; GHS, Global Health Status; BFI, Brief Fatigue Inventory; DT, Distress Thermometer; PDI, Psychological Distress Inventory; VRS, Verbal Rating Scale; CRF, Cancer-related fatigue; POMS, Profile of Mood States Questionnaire.

^a^ Effect Size (r): |0.1| ≤ r < |0.3| small effect; |0.3| ≤ r < |0.5| medium effect^x^; r ≥ |0.5| = large effect^xx^

NA = not applicable (Friedman Npar test with *p* > 0.05)

* *p* < 0.017

### Primary outcomes: Quality of Life indicators (EORTC-QLQ-C30)

Friedman’s non parametric test was significant with *p* < 0.001 for social functioning and general health scale; symptoms of fatigue and cognitive functioning were significant with *p* < 0.05. Only role and emotional functioning showed no significant intra-group differences.

Post hoc pairwise comparisons with Wilcoxon signed rank test and Bonferroni correction (*p* < 0.017) showed between which assessment time there was a significant difference in primary outcomes. The QoL indicators of social functioning, symptoms of fatigue, and general health scale were significantly different (*p* < 0.017) at T1 compared to T0, except cognitive functioning. Considering mean scores, analyses showed that participants’ social functioning ability worsened, but symptoms of fatigue decreased, and general health scale were improved.

At T2, analyses highlighted a worsening (*p* < 0.017) between mean scores of social functioning and an increase on the general health scale compared to T0, while the subscale concerning the symptoms of fatigue was not significant.

Regarding the T3, Wilcoxon signed rank test results were the same as the short-term pairwise comparison (T0-T1). Analyses showed a significant (*p* < 0.017) decrease in social functioning, and a significant improvement of fatigue symptoms and general health scale mean score.

### Secondary outcomes: Psychological health

Considering the secondary and exploratory psychological outcomes, Friedman’s non parametric test was significant with *p* < 0.001 for distress evaluation (DT), depression (POMS), anger (POMS), confusion (POMS), and strength/vigor (POMS). Also, fatigue (BFI, POMS), distress (PDI), cancer-related fatigue of verbal rating scale (VRS), and anxiety (POMS) with *p* < 0.05.

Post hoc pairwise comparisons using Wilcoxon signed rank test with Bonferroni correction (*p* < 0.017) showed that the domains concerning severity of fatigue (BFI), distress (DT), cancer-related fatigue (VRS), depression (POMS), and anger (POMS) were significantly (*p* < 0.017) improved at T1 assessment compared to T0. Distress (PDI), fatigue, confusion, strength/vigor, and anxiety (POMS) did not show a significance.

At T2 we observed better psychological conditions (*p* < 0.017) considering the perceived severity of fatigue (BFI), and all the POMS subscales (depression, anger, fatigue, confusion, strength/vigor) except anxiety. Likewise, pairwise comparisons between T0 and T3 highlighted an improvement (*p* < 0.017) in all domains, but no significant effects on strength/vigor (POMS).

## Discussion

The present contribution explored the psychological well-being of thirty Italian women treated for BC and was aimed at studying the efficacy of a home-based multidisciplinary lifestyle intervention, proposed at the beginning of the COVID-19 pandemic, on QoL indicators and psychological health over a 12-month follow-up period.

As broadly highlighted by literature [53, 54] coping with cancer and treatment-related issues means being at the side of a tricky and stressful process, which could lead to negative and long-lasting psycho-social impairment. In addition, patients with BC during the COVID-19 pandemic have been involved in a number of additional difficulties, from disruptions in routine health care to the dramatic reduction of support possibilities. Thus, for all these reasons, long-term QoL and psychological health issues in BC survivors during the pandemic period are of particular interest to personalize and optimize care [55].

The COVID-19 pandemic and restrictive measures have forced practitioners and cancer survivors to embrace modern technological solutions to maintain health care provision and support interventions [26, 56]. At the same time, some physical activity trials have been shifted to digital platforms and home-based protocols, showing encouraging results in the field [21, 57–59]. The findings of the present study suggest that the home-based adaptation of the MoviS protocol [31], among the other valid models proposed in the Italian context during the pandemic era, may help patients regain the best possible quality of daily life. Indeed, although the small sample size, our preliminary results highlighted the usefulness of the proposed home-based lifestyle intervention in improving psycho-social well-being in the short- and long- term, exploiting the potential of new approaches to support cancer patients throughout remote monitoring.

At baseline, participants rated their overall QoL as quite good (i.e., a mean score of 4.91 ± 1.03 on a seven-point scale from very poor to excellent condition), but at the same time they had low cognitive, emotional, social, and role skills (QoL indicators: primary outcomes). Contrary to what was expected considering the particular pandemic context and considering the measures concerning psychological health (secondary outcomes), women showed on average a non-alarming psycho-social status, even if they did not feel strong (i.e., strength/vigor subscale). In particular, variables concerning the severity of fatigue and mood states (i.e., depression, anger, fatigue, confusion, and anxiety) highlighted low scores, while the perception of distress and cancer-related fatigue were in the middle range. This can be justified by the fact that all participants were diagnosed and received surgery and treatments within the previous 12-month. Previous findings [54, 60] reported that there is a higher incidence of cancer-related fatigue after surgery or therapies due to distress and visible outcomes, which typically resolves in the year after the completion of medical treatments. However, in some cases (i.e., approximately 30%) patients could experience more persistent symptoms for up to ten years or more, establishing a condition of chronicity.

To explain the average non-suffering basal state of the sample, as previously mentioned, we had to widen our gaze to the bio-psycho-social components of psycho-physical health. Starting from the social, economic, and clinical features, most patients were in a couple relationship, were highly educated and worked independent, and received a quadrantectomy. These are characteristics that previous studies [61–63] identified as protective factors towards the perception of greater distress and powerlessness in coping with adverse life events such as cancer illness.

Moreover, a previous study [64] on oncological patients and a control group from the general population conducted in Italy 3-month after the beginning of the COVID-19 outbreak, stood out with interesting results to support our clinical data. Indeed, in this study [64], cancer patients seemed to be surprisingly less afraid, and psychologically healthier during the outbreak; this could be explained by the fact that since cancer is a frightening condition, coping and adaptive skills learned during the long-lasting care path could act as protective factors in case of other serious and potentially fatal risks (i.e., COVID-19 disease). In these regards, literature shows that in most cases cancer survivors are able to apply resilience mechanisms to manage stress in different situations [64, 65], and in the participants of our study this condition was powered by the continuous care and support by clinical and scientific experts.

The present study showed a significantly positive effect on the improvement of QoL indicators and psychological health at T1, T2 and T3, even if women did not show clinically significant levels of psychological discomfort at the beginning of the program.

Starting from the QoL indicators (primary outcomes), as desired, the most interesting result seems to be the achievement, at T1 and T3, of higher QoL levels (i.e., improvement in general health status and reduction of fatigue symptoms) compared to baseline. To our knowledge, there are no other studies in the Italian context that evaluated the QoL of BC survivors during a one-year follow-up in the first year of COVID-19 pandemic. Despite this, the sub-dimension concerning social interaction abilities showed an opposite trend and worsened during the intervention. However, to understand and to justify this outcome, we cannot ignore the environmental context and the evolution of the first year of the pandemic, characterized by increasingly stringent restrictions and lockdowns. Literature [66] on Italian samples with cancer during the first outbreak highlighted an increase in time spent on the Internet by patients, probably seeking information, and noticed general emotional, social, and relational difficulties. It is possible that the negative effects of isolation, the perception of alienation given by living in the same restricted ambience for a prolonged time, and the loss of daily activities have somehow conditioned relational stimulation.

To sum up, considering the primary outcomes, our findings only partially support the hypothesis 1, according to which we expect to verify an improvement in all dimensions at T1; however, long-term outcomes supported the hypothesis 2, in which we stated a stable situation over time (i.e., at T0-T1 and then at T0-T3 assessments).

According to the secondary and explorative outcomes, sedentary BC survivors—who engaged in our lifestyle program—experienced relief from their symptoms of distress, perception of the severity of general fatigue and cancer-related fatigue, depression, and anger, showing an increase in general psychological health after 3-month of scheduled exercises and proper nutritional advice. This results in line with a considerable body of research that recommends physical exercises as an effective tool for reducing mental health impairment and cancer-related psychological outcomes [67–70].

Our T2 assessment on secondary outcomes showed an improvement on fatigue, depression, and anger as evidenced in a short-term period, but a flattening to baseline for distress and cancer-related fatigue (as was the case for the SS-fatigue scale of QoL indicators). Recent longitudinal studies [54, 71] have tried to identify different trajectories for fatigue over time in BC survivors, however the course of fatigue is very complex: certain patients had high initial levels of cancer-related fatigue that declined over time, while others (< 20%) had low initial levels that later gradually increased. Moreover, it was identified an unclear interrelationship with psychosocial factors such as distress and sleep disturbance.

On the contrary, however, perception of confusion decreased during the lifestyle program and patients’ felt stronger. Finally, in line with our assumptions, the 12-month follow-up assessments showed a generalized enhancement in all psychological domains; strength did not improve, but anxiety scores significantly decreased compared to baseline, and the fluctuation of cancer-related fatigue at this time showed a significant improvement. This finding is strongly consistent with the most recent literature, in which oncological exercise represents an effective therapeutic intervention for the reduction of symptoms of anxiety and for improving QoL during and after treatments [72].

In summary, the results of secondary and exploratory outcomes of global psychological health only partially confirm hypothesis 1 and almost entirely support hypothesis 2, (i.e., stable situation at 12-month with T0-T1 pairwise comparison), but pointed out other effects, i.e., on fatigue mood state, confusion and anxiety (POMS), and distress (PDI), not immediately observable at T1.

In conclusion, the QoL and the global phycological health status significantly improved (although not in all domains), and this is an almost paradoxical outcome considering the general worsening of the QoL and well-being of the world population and Italian citizens in 2020, as highlighted by the scientific literature [70, 73–75].

Our preliminary study also provides a further opportunity for reflection and initial evidence to support the idea that home-based lifestyle interventions could be effective and alternative methods to support cancer survivors during the resumption of everyday life [76, 77]. Another key point in favor of our research also concerns the absence of dropouts between the start and the end of the 12-month follow-up, contrary to what tended to be highlighted by clinical trials, especially those delivered remotely for long periods of time [78–80].

We conducted this study in a moment of progressive isolation from daily human contact for the entire population. The sense of being part of a group care path, symbolized as an emotional belonging rather than a physical presence, probably played a decisive role in the adherence of the participants to the program. Moreover, these aspects, contextualized within the dramatic scenario of the first three months of lockdown in 2020, could also explain why the control arm scored the same as the intervention group at T1. As the patients enrolled for exercise, the controls were strongly supported remotely in their lifestyle habits (i.e., they received counseling and could access the DianaWeb portal to interact with an expert). We could suppose that the presence and availability of a health-care professional in a moment of global isolation and difficulties in receiving medical or specialist support and/or consultations have affected the results and have been major biases of the study. Alongside, this also led us to reflect on the importance of involving clinical professionals in the trials because they are able to maintain solid relationships within the group and use the potential of this network to better engage, involve, and motivate patients. To conclude, the current multidisciplinary home-based lifestyle intervention could be proposed as an *ad hoc* treatment in which the patients are engaged and supported, and they feel to be the protagonists despite the distance, the isolation, and the pandemic condition as well. According to the literature [81–84] these could be the crucial keys that prevented drop-out and contributed to high levels of participant satisfaction and mood improvement.

### Strengths and limitations

This is an emerging field of investigation, and our study provides preliminary results to shed light on the benefits of home-based lifestyle intervention in BC Italian survivors. To our knowledge, this is the first study in the Italian context that monitors the QoL of women diagnosed with BC during a 12-month follow-up period over a one-year of pandemic. In fact, we report the results of a one-year follow-up to allow for a better understanding of the short- and long-term effects of a lifestyle intervention based on psychological counseling, exercise, and proper nutrition. Furthermore, even if an adaptation of the lifestyle intervention has taken place due to the restrictions imposed during the COVID-19 pandemic, we focus on the approach adopted. Compared to other studies, our approach takes a holistic view in which the multidisciplinary team represents an important support in achieving and maintaining a correct lifestyle with the aim of improving QoL. In this regard, a strength of this work is its clinical significance. In fact, the results of this study help to advance the field by clarifying the importance of the multidisciplinary team in prescribing and monitoring a healthy lifestyle and by offering, albeit preliminarily, sufficient data to understand its applicability in the clinical and non-clinical settings, even outside the pandemic context.

However, some limitations should be taken into consideration. In fact, despite the results obtained, we are aware of the need to validate the presented intervention on a wider sample and through a more articulated and rigorous research design. In particular, a proper control arm (as foreseen by the original protocol of the present trial research, protocol: NCT 04818359 [31]) should be enrolled to have strong analyses. Moreover, the decision to collect data from a single institution might affect external validity of the findings; then, it would be appropriate to evaluate the possibility of stratifying the sample based on medical characteristics (e.g., tumor stage at diagnosis and type of surgery). Finally, we must consider that some measures included in this study consist of only one item (i.e., distress thermometer and verbal rating scale); this may have affected the results, although these scales are scientifically validated and the constructs (i.e., distress and cancer-related fatigue) measured by them have been widely evaluated in their different facets even by the other instruments considered for an overall health assessment.

## Conclusions

This is the first study exploring, in BC survivors, the effects of a lifestyle intervention based on exercise and proper nutrition on short- and long-term psycho-social well-being in the Italian pandemic context. Data from this study suggests that lifestyle interventions during follow-up after cancer diagnosis, as part of the adjuvant treatment for those patients, can improve global well-being and psychological health. Additionally, improvements in overall health can be achieved with lifestyle intervention, with results seen using home-based exercises and remote supervision.

Finally, further research is needed to: (i) investigate, through longitudinal and randomized controlled trials, the changes in HRQoL during the BC survival phase out of the pandemic scenario; (ii) integrate an evaluation from a relational perspective to understand which figures support the patients, their role, and the motivation they have to be eventually included in a similar intervention, which could also promote the caregiver’s and dyadic health [85].

## Supporting information

S1 Data Dataset study (N = 30). (XLSX)
